# Evaluation of the Acute Flaccid Paralysis Surveillance System in Polio-Free Jordan, 2012-2016: Retrospective Secondary Analysis

**DOI:** 10.2196/14217

**Published:** 2019-09-27

**Authors:** Fatima Zerriouh, Yousef Khader, Nabil Qasem, Kamel Abusal, Ibrahim Iblan, Layla Ghaffari, Mohammed Abdallat

**Affiliations:** 1 Jordan Field Epidemiology Training Program/Community Medicine Residency Program Jordan Ministry of Health Amman Jordan; 2 Department of Community Medicine Public Health and Family Medicine Faculty of Medicine, Jordan University of Science & Technology Amman Jordan; 3 Expanded Program on Immunization Communicable Diseases Directorate Jordan Ministry of Health Amman Jordan; 4 Jordan Field Epidemiology Training Program Jordan Ministry of Health Amman Jordan; 5 Communicable Diseases Directorate Jordan Ministry of Health Amman Jordan

**Keywords:** polio eradication, acute flaccid paralysis, surveillance, evaluation, Jordan, JFETP

## Abstract

**Background:**

As part of the polio-eradication strategy, the World Health Organization (WHO) has established a global acute flaccid paralysis (AFP) surveillance system. AFP surveillance has successfully helped Jordan achieve polio-free certification. However, there is a substantial risk of polio importation from neighboring countries including Syria and Iraq.

**Objective:**

This study aimed to evaluate the AFP surveillance in Jordan and identify areas that need improvement.

**Methods:**

This retrospective study is a secondary analysis of data that were routinely collected between 2012 and 2016 by Jordan’s Expanded Program on Immunization. The WHO’s minimum performance indicators were used to evaluate the AFP surveillance.

**Results:**

Cumulatively, 328 AFP cases had been reported. Almost half (n=168, 51.3%) of the patients were aged 1-5 years, and 55.8% (n=183) were male. All cases were discarded (classified as a nonpolio case). The most common cause of AFP was Guillain-Barre Syndrome (n=115, 35.1%). The annualized nonpolio AFP rate increased from 1.4/100,000 children below 15 years of age in 2012 to 4.3 in 2016. National and subnational sensitivities were not met in 2012 and 2013. Adequacy of stool specimens and timeliness of specimens arriving at and processed in the laboratory were constantly above the minimum target. Timeliness of the investigation met the expected target but with a decreasing trend. The nonpolio enterovirus isolation rate was below the target, except in 2016.

**Conclusions:**

The AFP surveillance system in Jordan is performing well; however, additional efforts are needed to strengthen the subnational sensitivity. The cold chain from sample collection to laboratory testing has to be maintained to ensure the reliability of stool specimens required for isolation of the nonpolio enterovirus.

## Introduction

In 1988, the 41st World Health Assembly adopted the Global Polio Eradication Initiative [1]. Since then, the number of poliomyelitis cases has decreased by over 99% in more than 125 endemic countries [2]. Four World Health Organization (WHO) regions—America, Western Pacific, Europe, and South-East Asia—were certified as polio-free in 1994, 2000, 2002, and 2014, respectively, and the disease was confined to three endemic countries (Nigeria, Pakistan, and Afghanistan) in 2014 [2]. Key strategies used by the Global Polio Eradication Initiative included strengthening childhood immunization through oral polio vaccines, conducting surveillance through investigation of AFP cases among children under 15 years of age, and conducting house-to-house “mop up” campaigns in areas where cases of polio have been identified [1,3].

Poliomyelitis is a highly contagious viral disease caused by any of three serotypes of the poliovirus, which belongs to the genus *Enterovirus* [4]. The virus is transmitted via the fecal-oral route, and humans are the only reservoir of the poliovirus. The virus affects mostly children under the age of 5 years. One of every 200 poliovirus infections results in clinically apparent paralytic disease. There is no cure for poliomyelitis; it can only be prevented via safe polio vaccination [2,4].

Acute flaccid paralysis (AFP) is defined as the sudden onset of weakness or paralysis of a limb, characterized as flaccidity (reduced tone), in a child younger than 15 years of age [5]. It is a complex clinical syndrome with several different etiologies including paralytic polio caused by wild poliovirus or circulating vaccine-derived poliovirus, Guillian-Barre syndrome (GBS), transverse myelitis, traumatic neuritis, meningitis, encephalitis, and brain tumors [6]. AFP surveillance includes detection and investigation of new-onset flaccid paralysis among children younger than 15 years of age or any other suspected poliomyelitis case among people of any age. It has been adopted globally as an essential strategy for monitoring the progress of the polio eradication initiative [7]. Nationwide AFP surveillance is essential to timely detect paralytic poliomyelitis due to wild poliovirus, to respond effectively to interrupt poliovirus transmission, to help monitor progress in polio eradication when polio exists in a country, to reveal the need of supplemental immunization activities, and to certify the absence of wild poliovirus circulation in countries with a polio-free status [7].

In Jordan, routine immunization against polio has been mandatory since 1979. The Polio Eradication Program led by the Jordan’s Expanded Program on Immunization (EPI) and endorsed by the WHO has successfully contributed to the decrease in poliomyelitis cases throughout the country and played a considerable role in attaining WHO polio-free certification for Jordan. Jordan reported the last indigenous polio case in 1988, although the last virologically confirmed polio case was reported on March 3, 1992, with the probable origin of the virus from Pakistan [8]. The EPI in Jordan has routinely collected AFP surveillance data since 1999 [9].

Polio outbreaks continue to occur in some countries [7]. Jordan is a neighboring country of Syria and embraces Syrian refugees. As such, it remains at risk of importation of polio, and it is the right time to evaluate the AFP surveillance activities to ensure that AFP surveillance is implemented with the required standards. Therefore, this study aimed to evaluate AFP surveillance in Jordan and describe its indicators according to WHO in 2012-2016 and to identify limitations and areas that need further improvement to maintain the polio-free status.

## Methods

### Study Design

This study was based on a secondary analysis of AFP surveillance data that were routinely collected between 2012 and 2016 by the EPI. All AFP cases reported to the EPI from all the 12 governorates and all health sectors during this period were included. In this study, the AFP case investigation form, laboratory investigation form, sample results, and 60-day follow-up data were evaluated. The WHO’s minimum performance indicators were used to evaluate the quality of AFP surveillance [5]. Official approval to conduct the study was obtained from the Ethical Committee at Jordan Ministry of Health.

### The Acute Flaccid Paralysis Surveillance System in Jordan

Jordan is divided into 12 governorates and 21 districts; the health system is represented in five health sectors: Ministry of Health, Royal Medical Services, Private Sector, Teaching Hospitals, and United Nations Relief and Works Agency. Approximately 37.3% of the population is under 15 years of age.

In the AFP surveillance system in Jordan, an AFP case is defined as any child below the age of 15 years who develops acute onset of flaccid paralysis (including GBS, transverse myelitis, or any other cause) or any suspected case of polio at any age [9]. Since October 2014, the WHO in Jordan has ensured that all AFP cases are notified and investigated as prospective polio cases immediately by a special team (WHO AFP medical officers), maintaining timeliness and completeness as advised by the WHO. When a patient meets the AFP case definition, the health care practitioners immediately notify (by telephone) the local public health officer who, with the assistance of the WHO AFP officer, conducts a comprehensive investigation using the standard WHO case investigation form that includes demographic information, clinical history, vaccination history, adequacy and time of stool specimen collection, and preliminary diagnosis. The public health officer also ensures collection of two stool specimens, 24-48 hours apart, within 14 days of symptom onset. The case investigation report is then sent to the EPI.

Active surveillance is conducted by the local public health officers once a week, and the WHO AFP officers follow a predefined schedule (3-4 times a week) to cover public and private sectors.

### Laboratory Investigation

Jordan’s national poliovirus laboratory in Amman is a WHO-accredited laboratory of the Eastern Mediterranean Region poliovirus laboratories network. It has routinely registered epidemiological and virological data from AFP surveillance since 1998 [9]. Jordan’s national poliovirus laboratory is equipped to isolate poliovirus from stool samples, identify poliovirus to confirm wild variety or circulating vaccine-derived poliovirus, and fulfill the examination of more than 150 contacts samples yearly. The collected specimens are sent to the WHO-accredited poliovirus isolation laboratory at the National central laboratory for enterovirus analysis. When there is a suspicion of polio, the sample is referred to the regional WHO-accredited laboratory to confirm the result and differentiate between the three poliovirus serotypes.

### Final Classification of Acute Flaccid Paralysis Cases

AFP classification in Jordan follows the WHO flow chart. An AFP case where two adequate stool specimens are analyzed and no poliovirus is isolated is classified as a nonpolio case (discarded). A case where the stool specimens are inadequate but the patient has no residual paralysis after 60 days of onset of symptoms is also classified as a nonpolio case (discarded). A case with inadequate stool specimens and residual paralysis after 60 days or one where the patient is lost to follow-up or dies within 60 days of symptom onset is referred to the National Polio Expert Committee for final classification (compatible with polio or should be discarded).

### Environmental Surveillance

The sampling and testing of sewage can identify poliovirus circulation in populations serviced by the sewage system and are used to complement AFP surveillance [9]. Environmental surveillance has been established in Jordan in November 2016 (Currently, three sites in three governorates including at Zaatari camp, one governorate at the Syrian borderline, and the Amman governorate [most populated]) [9].

### Data Analysis

Data were analyzed using a data management system for AFP surveillance data—Information for Action (Version 4. Geneva, Switzerland: World Health Organization) and Excel 2010 (Redmond, WA: Microsoft Corp). Descriptive analysis was used to describe the epidemiology of AFP in Jordan and to calculate statistics based on the standard WHO performance indicators for AFP surveillance.

## Results

### Overview

**Figure 1:**
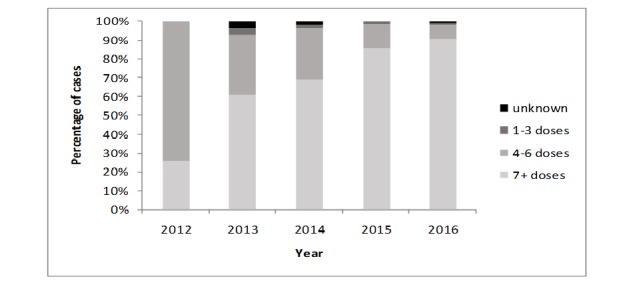
Percentage of polio-vaccinated acute flaccid paralysis cases in Jordan between January 2012 and December 2016.

Cumulatively, 328 AFP cases were reported to the EPI between January 2012 and December 2016. There were two hot cases: one in Balqa governorate in 2012 and another in Mafrak in 2013. All cases were discarded. Of all cases, 168 (51.3%) were of patients between the ages of 1 and 5 years, and 183 (55.8%) were male. Almost half (163, 49.7%) of all cases had fever, 108 cases (32.9%) had asymmetric paralysis, and 113 cases (34.5%) had rapid progression of paralysis (Table 1). Of all cases, 292 (89.0%) had known polio immunization status. Figure 1 shows the immunity profile of AFP cases by year. Vaccination coverage of more than seven doses of oral polio vaccine was as high as 77.1% (n=253) of all AFP cases reported (Figure 1).

### Classification of Acute Flaccid Paralysis Cases

**Figure 2:**
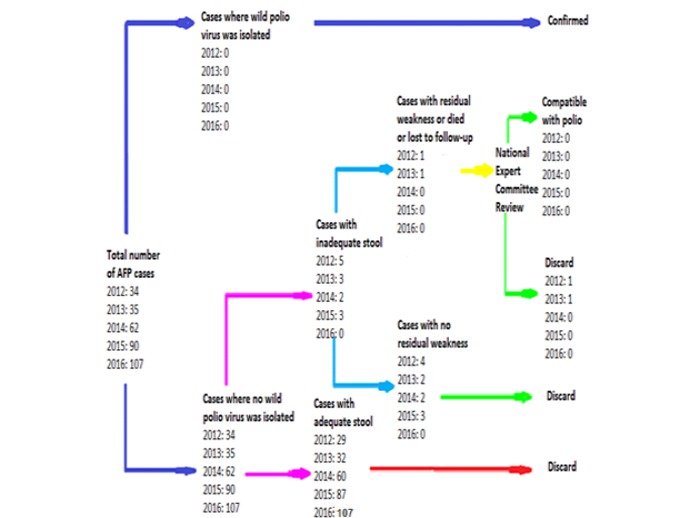
Flow chart showing the virological classification of AFP cases reported in Jordan between 2012 and 2016.

The AFP cases were classified according to the WHO virological classification flowchart (Figure 2). A total of 13 cases (4%) had inadequate specimens and were reviewed by the National Polio Expert Committee (NPEC) who classified them as “discarded.” A variety of diagnoses were identified as causes of AFP. The most common causes were GBS (115, 35.1%), myositis (49, 14.9%), encephalitis (14, 4.3%), and transverse myelitis (14, 4.3%).

**Table 1:** Descriptive epidemiology of 328 acute flaccid paralysis cases reported in Jordan between January 2012 and December 2016.

Characteristic		Value, n (%)
**Sex**		
	Male	183 (55.8)
	Female	145 (44.2)
**Age (years)**		
	<1	34 (10.4)
	1-5	168 (51.3)
	6-10	88 (26.8)
	11-15	38 (11.5)
**Clinical symptoms**		
	Fever	163 (49.7)
	Asymmetric paralysis	108 (32.9)
	Rapid progression of paralysis	113 (34.5)

### Evaluation Outcomes

Jordan’s cumulative annualized nonpolio AFP rate was 2.5 AFP cases per 100,000 people below the age of 15 years per year in 2012-2016. The country’s AFP incidence rate increased from 1.4 cases in 2012 to 4.3 cases per 100,000 people below the age of 15 years in 2016 (Table 2). Analyzing AFP rates by governorate showed that during 2014-2016, all governorates fulfill the expected rate except three (Karak, Maan, and Madaba), which were silent in 2014, and the majority of governorates failed to consistently surpass the WHO minimum target of 2 AFP cases per 100,000 people under the age of 15 years in 2012 and 2013 (Table 3).

**Table 2:** Acute flaccid paralysis surveillance performance indicators for Jordan, 2012-2016.

Indicators of surveillance performance	Target	Country performance by year				
		2012	2013	2014	2015	2016
Percentage of all expected monthly reports received	≥90	100	100	100	100	100
Annualized nonpolio AFP^a^ rate per 100,000 children under 15 years of age	≥2	1.4	1.4	2.3	3.2	4.3
Percentage of AFP cases investigated within 48 hours	≥80	100	100	98	98	95
Percentage of AFP cases with two adequate stool specimens collected 24-48 hours apart and ≤14 days after onset	≥80	85	91	97	97	100
Percentage of specimens arriving at the laboratory in good condition	≥80	100	100	100	100	100
Percentage of specimens arriving at a WHO-accredited laboratory within 3 days of being sent	≥80	100	97.1	95.2	94.4	98.6
Percentage of specimens for which laboratory results were sent within 28 days of receipt of specimens	≥80	97	100	100	100	96
Nonpolio enterovirus isolation rate of stool specimens submitted to the laboratory having nonpolio enterovirus isolated (%)	≥10	3	6	6	6	10
Percentage of AFP cases requiring a follow-up examination that were examined at 60 days after the onset of paralysis	≥80	94	94	97	90	95

**Table 3:** Incidence rate of annualized nonpolio acute flaccid paralysis per 100,000 children below the age of 15 years per year and governorate in Jordan in 2012-2016. The World Health Organization minimum target is ≥2 cases.

Governate	2012	2013	2014	2015	2016
Ajloun	1.7	0.0	4.9	3.2	4.0
Amman	1.5	1.6	2.6	2.0	3.0
Aqaba	2.0	0.0	1.9	4.2	5.0
Balqa	0.6	1.3	1.8	3.0	3.3
Irbid	1.2	1.8	3.1	2.0	2.2
Jarash	2.6	1.3	3.7	5.7	4.7
Karak	3.2	1.2	0.0	4.0	5.7
Maan	2.1	0.0	0.0	4.3	7.4
Madaba	0.0	1.6	0.0	2.1	3.3
Mafrak	0.8	3.3	3.1	3.0	4.1
Tafileh	0.0	2.6	5.0	3.3	6.1
Zarqa	1.6	1.6	2.0	2.1	2.7

Table 2 shows the AFP surveillance performance indicators for Jordan in 2012-2016. The percentage of AFP cases with two adequate stool specimens was constantly above the minimum target of ≥80% in all governorates and reached 100% in 2016. The percentages of specimens arriving at the laboratory within 3 days of being sent were constantly above the WHO minimum target of at least 80%. The proportions of specimens processed in the laboratory within 28 days of specimens receipt were above the WHO minimum target of at least 80% during the time period study. More than 90% of the 328 AFP cases reported were investigated within 48 hours of being notified, which is above the WHO minimum target of at least 80%. The proportion of patients notified within 7 days of onset of symptoms was above 80%. All AFP cases requiring 60-day follow-up were examined at 60 days of onset of symptoms, reaching the expected target. The proportions of stool specimens where nonpolio enterovirus was isolated were below the WHO minimum target of at least 10%, except in 2016 when it reached the target; however, these proportions increased from 3% in 2012 to 6% in 2013, 2014, and 2015.

## Discussion

### Principal Findings

This study evaluated the Jordan AFP surveillance performance over a 5-year period (2012-2016). Jordan is one of the many countries to attain polio-free certification. This study showed that there was no wild poliovirus isolated or any compatible case classified by the NPEC. However, importation of poliovirus remains a potential threat; therefore, it is essential to continue AFP surveillance. The study reported that half of the reported cases were of patients below 5 years of age, which is consistent with findings of a study conducted in Iran [10] and another one in Bangladesh [11]. However, higher percentages were reported in countries such as Congo (85.2%) [12] and Ghana (74.4%) [13], and lower percentages were reported in other countries including Italy (37%) [14]. Of all cases, the number of boys exceeded the number of girls, a finding also reported in other studies [11,13,14].

Almost half of the patients (49.7%) had fever, and approximately one-third of the patients had asymmetric paralysis. This finding is similar to that in Iran [10]. Odoom et al [13] reported that 84.2% of cases had fever and 64.8% had asymmetric paralysis, and another study in Turkey reported that 13.6% of the total cases had fever [15].

Vaccination coverage of more than seven doses of oral polio vaccine was as high as 77.1% of all AFP cases reported. This impressive vaccination coverage is explained by the repetitive campaigns conducted in 2013-2016 that targeted prevention of polio importation from Syrian refugees and the high awareness of parents to ensure completion of the vaccination schedule and taking recommended doses during campaigns. A similar finding was revealed in Bangladesh (75%) [11]. In Turkey, 84.5% of the studied cases had at least one oral polio vaccine dose [15].

GBS was the most common cause of AFP and found in 35.1% cases in Jordan. A much higher rate (85.4%) was reported in a study in Iran [10]. In South Africa, in 2013, 42.7% of the AFP cases were caused by GBS [16]. In addition, >50% of AFP cases caused by GBS reflects the quality of AFP surveillance. Increased awareness of pediatricians and neurologists regarding the importance of reporting any suspected GBS will help identify a considerable number of AFP cases, since GBS was found to be the most common diagnosis of AFP in Jordan and other countries [12,17].

The sensitivity of the AFP surveillance system is reflected by the annualized nonpolio AFP rate. This study showed that the overall annualized nonpolio AFP rate (2.5/100,000 people below 15 years of age) exceeded the WHO target. Despite the increasing trend of the annualized nonpolio AFP rate over the 5-year study period, the AFP surveillance system failed to reach the minimum WHO requirement in 2012 and 2013. However, it is worth mentioning that Jordan performed well in fulfilling the WHO target in 2014, 2015, and 2016, with 2.3, 3.2, and 4.3 cases per 100,000 people below 15 years of age, respectively. This is explained by establishment of the WHO AFP officer team in 2014, which has since strengthened the active AFP surveillance. Subnationally, the annualized nonpolio AFP rate revealed that even in 2014, the expected target was not met for certain governorates (Karak, Maan, and Madaba) that were totally silent.

The percentage of AFP cases with two adequate stool specimens collected 24-48 hours apart and ≤14 days after onset is another surveillance performance indicator and should be ≥80%. Our study showed that Jordan performed well in meeting this target constantly over the 5-year period, with an increasing trend up to 100% in 2016. This reflects the importance of the early detection of AFP cases. The timeliness of investigation of AFP cases reported exceeded the WHO target from 2012 to 2016. However, there is an alarmingly decreasing trend that can be explained by the increase of AFP cases and investigation over the study period. Jordan has to maintain the performance well over time, and therefore, there is a need for periodic sensitization of public health officers and the WHO AFP officer team with regard to the importance of maintaining the investigation of AFP cases within 48 hours. This study showed that all specimens that arrived at the laboratory were in good condition, but the results are combined with those of the stool specimen adequacy tests, as there were no separate data for the latter. The percentage of specimens arriving at a WHO-accredited laboratory within 3 days of being sent also reflects the timeliness of the process. The AFP surveillance system succeeded in achieving the expected WHO target from 2012 to 2016. Jordan’s national poliovirus laboratory performed well in accomplishing the timeliness of AFP surveillance system; this is may be due to the proximity and the centrality of Jordan’s national poliovirus laboratory from the governorates and to the effort made by the EPI and the staff of the Jordan’s national poliovirus laboratory. The evaluation of the viability of stool specimens sent to the laboratory is represented by the nonpolio enterovirus isolation rate, which should be at least 10% and was not achieved by the AFP surveillance system during the study period; this indicates that the reverse cold chain was not maintained in that period, except in 2016 where it was in line with the WHO target. A high nonpolio enterovirus isolation rate was reported in Ghana [13], Bangladesh [11], and Turkey [15]. More than 90% of the AFP cases reported during the study period and requiring 60-day follow-up examination had been examined at 60 days after the onset of paralysis, meeting the specified WHO target. Furthermore, when there is no need of the NPEC review, the EPI takes a simple random sample from the annual AFP cases to the NPEC to ensure that the AFP surveillance is functioning well.

### Conclusions

The Jordan AFP surveillance system is performing well in meeting and exceeding the WHO targets. However, national performance can obscure the subnational performance and prevent early detection of AFP cases, which can occur at the district level. Therefore, subnational surveillance has to meet the WHO targets in a disaggregated way. Moreover, the cold chain from sample collection to laboratory testing has to be maintained to ensure the reliability of stool specimens required for the isolation of nonpolio enteroviruses. Environmental surveillance is another strategy for maintaining a polio-free status; Jordan has this strategy in place, but more areas need to be selected to cover the whole country. Sharing polio experiences between countries is advisable to meet the global polio eradication goals.

## Abbreviations

AFP: Acute Flaccid Paralysis
EPI: Expanded Program on Immunization
GBS: Guillain-Barre Syndrome
NPEC: National Polio Expert Committee
WHO: World Health Organization
